# Simultaneous detection of *Plasmodium vivax* and *Plasmodium falciparum* gametocytes in clinical isolates by multiplex-nested RT-PCR

**DOI:** 10.1186/1475-2875-11-190

**Published:** 2012-06-10

**Authors:** Napaporn Kuamsab, Chaturong Putaporntip, Urassaya Pattanawong, Somchai Jongwutiwes

**Affiliations:** 1 Molecular Biology of Malaria and Opportunistic Parasites Research Unit, Department of Parasitology, Faculty of Medicine, Chulalongkorn University, Bangkok, 10330, Thailand

**Keywords:** Malaria diagnosis, Gametocyte, Reverse transcription polymerase chain reaction, *Plasmodium falciparum*, *Plasmodium vivax*, *Pfs25*, *Pvs25*

## Abstract

**Background:**

Gametocyte carriage is essential for malaria transmission and endemicity of disease; thereby it is a target for malaria control strategies. Malaria-infected individuals may harbour gametocytes below the microscopic detection threshold that can be detected by reverse transcription polymerase chain reaction (RT-PCR) targeting gametocyte-specific mRNA. To date, RT-PCR has mainly been applied to the diagnosis of *Plasmodium falciparum* gametocytes but very limited for that of *Plasmodium vivax*.

**Methods:**

A multiplex-nested RT-PCR targeting *Pfs25* and *Pvs25* mRNA specific to mature gametocytes of *P. falciparum* and *P. vivax*, respectively, was developed. The assay was evaluated using blood samples collected in rainy and dry seasons from febrile patients,in a malaria-endemic area in Thailand. Malaria diagnosis was performed by Giemsa-stained blood smears and *18S rRNA* PCR.

**Results:**

The multiplex-nested RT-PCR detected *Pfs25* mRNA in 75 of 86 (87.2%) *P. falciparum*-infected individuals and *Pvs25* mRNA in 82 of 90 (91.1%) *P. vivax* malaria patients diagnosed by *18S rRNA* PCR. Gametocytes were detected in 38 (eight *P. falciparum* and 30 *P. vivax*) of 157 microscopy positive samples, implying that a large number of patients harbour sub-microscopic gametocytaemia. No seasonal differences in gametocyte carriage were observed for both malaria species diagnosed by multiplex-nested RT-PCR. With single-nested RT-PCR targeting *Pfs25* or *Pvs25* mRNA as standard, the multiplex-nested RT-PCR offered sensitivities of 97.4% and 98.9% and specificities of 100% and 98.8% for diagnosing mature gametocytes of *P. falciparum* and *P. vivax,* respectively. The minimum detection limit of the multiplex-nested PCR was 10 copies of templates.

**Conclusions:**

The multiplex-nested RT-PCR developed herein is useful for simultaneous assessment of both *P. falciparum* and *P. vivax* gametocyte carriage that is prevalent and generally sympatric in several malaria-endemic areas outside Africa.

## Background

Gametocytes, the precursors of male and female gametes, of malaria parasites are formed in the human host through the developmental switch from asexual replication in erythrocytes. Although gametocytes are not responsible for clinical symptoms, they ensure the transmission of malaria to another host. Upon taking a blood meal, gametocytes are transferred to a mosquito’s midgut lumen where they differentiate into male and female gametes. After complete sexual reproduction and successive processes of sporogonic development, mature sporozoites accumulate in the vector’s salivary gland, ready to be inoculated into a new host. Therefore, the presence of gametocytes in circulation of infected individuals is imperative for malaria to remain endemic in a given community. Malaria control strategies aiming at interruption of the malaria transmission process require knowledge on the status of gametocyte carriage in each endemic area [1].

During acute malaria infection, the number of gametocytes in circulation of patients occurs at much lower densities than asexual stages and usually circulates at the level close to or below microscopic detection threshold, making it liable to be undiagnosed by microscopy [2,3]. Several epidemiological surveys have shown that only a subset of malaria-infected individuals possessed gametocytes upon microscopic examination of the blood smears [3-7]. Importantly, these gametocyte-negative blood samples remain infective to anopheline vectors akin to those that have patent gametocytaemia [6-8]. On the other hand, molecular detection of *Plasmodium* gametocytes has revealed that a considerable number of malaria patients whose blood samples were gametocyte-negative by microscopy actually had sub-microscopic gametocytaemia [3-7]. Therefore, microscopy detection of gametocytes could underestimate and thereby mislead evaluation of malaria transmission potential in endemic areas.

Molecular diagnostics of malarial gametocytes are based on amplification of mRNA transcripts that are exclusively expressed during gametocyte stages. Some of these transcripts are synthesized in co-ordination with specific periods of gametocyte development while some are sex-specific [9,10]. Therefore, specific mRNA transcripts could serve as appropriate markers for diagnosing stages of gametocytes. Of these, transcription of *Pfs25* begins when gametocytes of *Plasmodium falciparum* become mature (stage V) and continues until the formation of ookinetes [11]. Importantly, homologues of *Pfs25* have been identified in several other malaria, e.g. *Pvs25, Pbs25, Pgs25* and *Pys25* in *Plasmodium vivaxPlasmodium berghei, Plasmodium gallinaceum* and *Plasmodium yoelii,* respectively [12]*.* To date, molecular epidemiological studies have been largely performed to assess *P. falciparum* gametocyte carriage because it is the most prevalent and most pernicious malaria species that requires urgent effective control measures. On the other hand, a few reports have demonstrated molecular application for diagnosing *P. vivax* gametocytes [5,13] despite the fact that it is the most widely distributed species with relapsing potential and is responsible for an important global public health burden [14].

Both *P. falciparum* and *P. vivax* are major malaria species and sympatric in several endemic areas outside Africa [15]. Therefore, epidemiological surveillance or assessment of malaria transmission by estimation of both *P. falciparum* and *P. vivax* gametocyte carriage in each endemic area is essential for malaria control policy. Because the expense of reagents and turnaround time in multiplex PCR are less than single PCR, a multiplex-nested RT-PCR assay targeting *Pfs25* and *Pvs25* is developed for rapid detection and differentiation of *P. falciparum* and *P. vivax* gametocytes simultaneously. Diagnostic performance of the method was evaluated using blood samples collected during both high and low transmission seasons from malaria patients in an endemic area of Thailand.

## Methods

### Clinical sample collection and study area

Blood samples (~1 mL) were collected from 235 febrile patients (136 males and 99 females; mean age 21.7 years, range seven to 77 years) who attended a malaria clinic at Umpang District in Tak Province (GPS N16° 1′ 0″, E98° 51′ 46″), north-western Thailand bordering Myanmar, during the rainy season (from June to August 2010) and the dry season (from November 2010 to January 2011). All except one patient had febrile onset one day prior to blood sample collection. In total, 157 patients had malaria in their circulation based on microscopic diagnosis of Giemsa-stained blood smears. Malaria was not found by microscopy in the remaining 78 febrile individuals. Each blood sample was divided and preserved separately in EDTA and RNA*later*^TM^ (Ambion, USA). Exclusion criteria were those having previous anti-malarial treatment or presence of clinical signs and symptoms of severe malaria [16]. Informed consent was obtained from each patient or from a parent or guardian for those aged less than 18 years. The ethical aspects of this study have been approved by the Institutional Review Board of Faculty of Medicine, Chulalongkorn University.

### Microscopy

Both thin and thick blood smears were prepared from each patient and stained with Giemsa solution for microscopic diagnosis of malaria species and stages. Parasite density was assessed from a thick blood film for ≥200 leukocytes with a 100× objective. Estimation of parasite density was performed by assuming a standard count of 7,000 leucocytes/μL [17]. Each stained slide was examined independently by two microscopists who were blinded to each other results. The mean values of parasite density were used for further analysis.

### DNA and RNA extraction

DNA was extracted from 200 μL of EDTA-preserved blood samples using QIAGEN kit following the instruction protocol except for elution with 30 μL TE buffer. Blood samples (200 μL) preserved in RNA*later*^TM^ were used for RNA extraction using QIAamp RNA blood mini kit (Qiagen, Germany) and eluted with 30 μL of RNase-free water. cDNA was generated from two μL of each RNA sample and amplified by using Takara RNA PCR kit (AMV) version 3.0 (Takara, Japan) in a total volume of 10 μL. Two μL of RNA products of each sample were used as template during subsequent PCR assay to exclude possible genomic DNA contamination in cDNA templates.

### Diagnosis of malaria species by nested PCR

Nested PCR targeting the small subunit ribosomal RNA gene (*18S rRNA* PCR) of all four human malaria species and*. Plasmodium knowlesi* was done following protocol and amplification conditions as described previously [18,19]. Results were obtained from 2% agarose gel electrophoresis, stained with ethidium bromide solution and visualized under UV transillumination.

### Positive and negative controls

Positive controls for *Pfs25* were genomic DNA and cDNA of a clinical isolate that contained 315 mature *P. falciparum* gametocytes/μL. For *Pvs25*, genomic DNA and cDNA of a *P. vivax* clinical isolate harbouring 385 mature *P. vivax* gametocytes/μL were used as positive controls. Verification of single species infection in these control samples was done by using *18S rRNA* PCR. Sterile water was used as negative control.

### Multiplex-nested RT-PCR targeting *Pfs25* and *Pvs25*

Primers for primary PCR (primers FV25F0 and FV25R0) were used to amplify both *Pfs25* and *Pvs25* and those for secondary PCR were specific to each *Plasmodium* species (Table 1). Secondary PCR was performed using primers F25F1 and F25R1 specific for *Pfs25* and primers V25F1 and V25R1 specific for *Pvs25* in the same reaction tubes. Optimization of PCR was performed by adjustment of thermal cycler profiles and concentration of primers to obtain a final condition that provided good intensities for both amplicons without non-specific bands. The optimal DNA amplification was carried out in a total volume of 20 μL containing 2 μL of cDNA template, 2.5 mM each deoxynucleotide triphosphate, 2 μL of 10x PCR buffer, 1.6 μL of 25 mM MgCl_2_, 0.08 μL of 30 μM of each primer for primary PCR and 0.4 units of rTaq DNA polymerase (Takara, Seta, Japan). Thermal cycler profile for primary PCR contains 94°C for 1 m for one cycle; 94°C for 40 s, 55°C for 30 s and 72°C for 30 s for 25 cycles; and 72°C for 5 m for one cycle. Secondary PCR contained similar reaction mixtures except that 0.06 μL of 30 μM of each secondary PCR primer and 1 μL of primary PCR product as template were used. A total of 26 amplification cycles were used for secondary PCR. The amplification products were analysed by 2% agarose gel electrophoresis.

**Table 1:** **PCR primers used for amplification of*Pfs25*and*Pvs25***

**Gene target**	**Primers**	**Sequences (5′ → 3′)**	**Nucleotide positions**	**Product size (bp)**
Primary PCR				
*Pfs25* and *Pvs25*	FV25F0	GAAGATACATGTGAAGAAAAA	237–257* or 163–183#	264 for *P. falciparum*
	FV25R0	ATTGGGAACTTTGCCAATA	482–500* or 414–432#	270 for *P. vivax*
Secondary PCR				
*Pfs25*	F25F1	AAATGTGACGAAAAGACTG	264–281*	201
	F25R1	AGTTTTAACAGGATTGCTTGTATC	441–464*	
*Pvs25*	V25F1	ACCCTAGGCAAAGCATG	202–218#	115
	V25R1	CAAGTGTCTTCCTTCAAAGT	298–317#	

* and # after GenBank^TM^ accession numbers X07802 and GU256271 for *Pfs25* and *Pvs25*, respectively.

### Detection of *Pfs25* and *Pvs25* by single-nested RT-PCR

The PCR primers and amplification conditions for single-nested RT-PCR were essentially the same as those for multiplex-nested RT-PCR except that secondary PCR was performed in separate reaction tubes for each pair of primers. The number of amplification cycles for primary PCR and secondary PCR were essentially the same as single-nested PCR assays.

### Detection limit and specificity

To estimate the detection limit of the developed methods, the entire *Pfs25* using primers PFS25F0, 5′-ATGAATAAACTTTACAGTTTG-3″ (nucleotides 75–95, positions after GenBank^TM^ accession no. X07802) and PFS25R0, 5′-TTACATTATAAAAAAGCATACTG-3′ (nucleotides 706–728) was amplified by PCR. Amplification condition contained 94°C for 1 m; 35 cycles of 94°C for 40 s, 53°C for 30 s and 72°C for 30 s; and 72°C for 5 m. Likewise, the entire *Pvs25* was amplified using primers PVS25F0, 5′-ATGAACTCCTACTACAGCCTC-3′ (nucleotides 1–21, positions after GenBank^TM^ accession no. GU256271) and PVS25R0, 5′-TTATATGACGTACGAAGGGAC-3′ (nucleotides 640–660) with the same amplification conditions. The PCR products of *Pfs25* and *Pvs25* comprised 654 and 660 bp (henceforth *Pfs25*L and *Pvs25*L), respectively, equivalent to 6.88 × 10^−7^ and 6.94 × 10^−7^ pg; thereby the copy number of each DNA template could be calculated. After the PCR-amplified products were gel purified, the concentration of each template was determined using the NanoDrop apparatus (Thermo Fisher Scientific, Delaware, USA). The dilutions of 1 × 10^6^, 1 × 10^5^, 1 × 10^4^, 1 × 10^3^, 1 × 10^2^, 10, 1 and 0.1 copy/μL were used as templates for determination of the detection limit of each gene target. To test whether co-existence of *P. falciparum* and *P. vivax* could influence sensitivity of each PCR assay, artificially mixed DNA templates from both malaria species with substantial difference in copy numbers were assessed. Specificity of PCR assays was analysed against genomic DNA (five isolates each) of *Plasmodium malariae, Plasmodium ovale* and *P. knowlesi*. The nested RT-PCR method targeting *Pfs25* with primers Pfs25-1, Pfs25-2, Pfs25-3 and Pfs25-4 developed by Babiker and colleagues was also used for evaluation [20].

### Data analysis

Diagnostic performance for multiplex-nested RT-PCR was evaluated with the results from single-nested RT-PCR assay of the same cDNA templates as the gold standard. Performance indices were the number of true positive (TP), number of true negative (TN), number of false positive (FP) and number of false negative (FN). Sensitivity was expressed as TP/(TP + FN) and specificity as TN/(TN + FP). The likelihood ratios for a positive test result were calculated as sensitivity/(1 – specificity), and for a negative test result as (1 – sensitivity)/specificity [21]. Differences in parasite densities between gametocyte-positives and gametocyte-negatives by microscopy were calculated by using the Mann–Whitney U test. A 2-tailed *p* value of <0.05 is considered significance.

## Results

### Detection limits of single-nested RT-PCR for *Pfs25* and *Pvs25*

**Figure 1:**
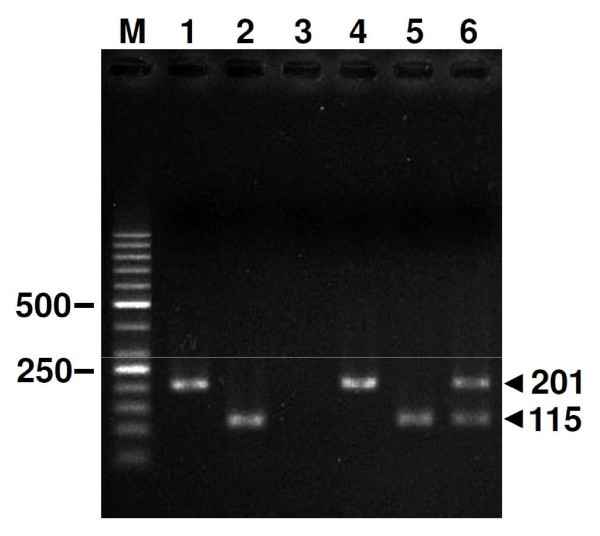
**Multiplex nested RT-PCR targeting*Pfs25*and*Pvs25*.** Representative 2% agarose gel electrophoresis showing specific amplifications generated from secondary PCR. Lane M, 50-bp ladder marker; lanes 1–6, *P. falciparum* genomic DNA, *P. vivax* genomic DNA, water as negative control, *P. falciparum* cDNA*, P. vivax* cDNA and mixed *P. falciparum* and *P. vivax* cDNA, respectively. Molecular size in base pairs is shown on the left and right of the gel.

Single-nested PCR targeting *Pfs25* and *Pvs25* could amplify genomic DNA of *P. falciparum* and *P. vivax* generating products of expected sizes, i.e. 201 and 115 bp, respectively (Figure 1). Likewise, concordant results were obtained from cDNA templates generated from mRNA of these parasite species. The minimum dilutions of either *P. falciparum* or *P. vivax* genomic DNA that gave reproducible positive results were 10 copies. Neither PCR assays exhibited cross-reactivity with genomic DNA from non-matched malaria species, indicating specificity of these tests. Meanwhile, a minimum of 100 copies was required for successful amplification of *Pfs25*L by the RT-PCR method developed by Babiker and colleagues [20].

### Detection limits of multiplex-nested RT-PCR

The multiplex-nested RT-PCR developed herein gave single 201-bp product for *P. falciparum* and 115-bp product for *P. vivax,* identical to single-nested PCR targeting each gene*.* Neither cross-hybridization nor false amplification between primers for *Pfs25* and *Pvs25* was observed when both malaria DNA were used as templates. The minimum detection limits were 10 copies of template DNA and the results were reproducible in five assays with different DNA templates. The sensitivity remained unaltered when numerous amounts of DNA templates from the other malaria species were added in the reactions, e.g. 10 copies of *P. vivax* DNA mixed with 1 x 10^6^ copies of *P. falciparum* DNA (Table 2). Results were essentially the same when cDNA of different isolates was used as templates.

**Table 2:** **Detection of*Pfs25*and*Pvs25*in artificial mixed DNA templates by single nested RT-PCRs and multiplex nested RT-PCRs**

**DNA templates (no. of copy)**	**Single nested RT-PCR**		**Multiplex nested RT-PCR**	
	**Pfs25**	**Pvs25**	**Pfs25**	**Pvs25**
Pfs25L (0.1) + Pvs25L (10^6^)	−	+	−	+
Pfs25L (1) + Pvs25L (10^6^)	−	+	−	+
Pfs25L (10) + Pvs25L (10^6^)	+	+	+	+
Pfs25L (10^2^) + Pvs25L (10^6^)	+	+	+	+
Pfs25L (10^5^) + Pvs25L (10^6^)	+	+	+	+
Pfs25L (10^6^) + Pvs25L (10^5^)	+	+	+	+
Pfs25L (10^6^) + Pvs25L (10^2^)	+	+	+	+
Pfs25L (10^6^) + Pvs25L (10)	+	+	+	+
Pfs25L (10^6^) + Pvs25L (1)	+	−	+	−
Pfs25L (10^6^) + Pvs25L (0.1)	+	−	+	−

### Microscopy and *18S rRNA* PCR diagnosis

Malaria was microscopically diagnosed in 157 of 235 febrile patients. Most patients were infected with either *P. falciparum* (n = 76) or *P. vivax* (n = 80). Co-infection with both species was observed in a patient. Besides asexual blood stages, *P. falciparum* gametocytes were observed in eight isolates (8.9% of total *P. falciparum*-positives by *18S rRNA* PCR) and *P. vivax* gametocytes in 30 isolates (31.1% of total *P. vivax*-positives by *18S rRNA* PCR). Meanwhile, *18S rRNA* PCR gave concordant results with microscopy in 215 blood samples. Microscopy under-diagnosed 11 out of 12 patients who had co-infections with ≥2 malaria species (Table 3). Furthermore, eight microscopy-negative samples were positive for *P. falciparum* (n = 7) or *P. vivax* (n = 1).

**Table 3:** Microscopy and molecular diagnosis of malaria species and gametocytes in 235 clinical isolates

**Infection**	**Microscopy**	***18S rRNA* PCR**	**Single nested RT-PCR**		**Multiplex nested RT-PCR**	
			**Pfs25**	**Pvs25**	**Pfs25**	**Pvs25**
Monoinfection						
*P. falciparum*	76	75	−	−	−	−
Gametocyte	8	−	68	−	67	−
*P. vivax*	80	78	−	−	−	−
Gametocyte	30	−	−	74	−	74
Co-infections						
*P. falciparum* and *P. vivax*	1	10	−	−	−	−
Gametocyte	0	−	9	6	8	6
*P. vivax* and *P. malariae*	0	1	−	−	−	−
Gametocyte	0	−	0	1	0	1
*P. falciparum*, *P. vivax*	0	1	−	−	−	−
and *P. malariae*						
Gametocyte	0	−	0	1	0	1
Total malaria positive cases	157	165	−	−	−	−
Total *P. falciparum*	77	86	−	−	−	−
Gametocyte (%)*	8 (8.9)	−	77 (89.5)	−	75 (87.2)	−
Total *P. vivax*	81	90	−	−	−	−
Gametocyte (%)**	30 (31.1)	−	−	82 (91.1)	−	82 (91.1)

* Percentage of *P. falciparum* gametocyte positives per total *P. falciparum* positives by *18S rRNA* PCR.

** Percentage of *P. vivax* gametocyte positives per total *P. vivax* positives by *18S rRNA* PCR.

Dashes indicate no applicable data.

### Diagnostic performance of *Pfs25* and *Pvs25* mRNAs in clinical samples

Single-nested RT-PCR targeting *Pfs25* gave positive results in 77 blood samples (89.5% of total *P. falciparum* positives by *18S rRNA* PCR) whereas 82 samples were positive for *Pvs25*, indicating the presence of mature gametocytes in these patients (91.1% of total *P. vivax* positives by *18S rRNA* PCR). No amplification occurred when RNA of these isolates collected prior to cDNA synthesis was added in the assays, indicating no genomic DNA contamination. Results inferred from multiplex-nested RT-PCR revealed that *P. falciparum* and *P. vivax* gametocytes co-existed in four of 11 patients who were co-infected with both these malaria species (diagnosed by *18S rRNA* PCR). Likewise, similar results were obtained when multiplex-nested RT-PCR was applied to the same cDNA samples (87.2% and 91.1% for *P. falciparum* and *P. vivax*, respectively) except that single-nested RT-PCR targeting *Pfs25* yielded two more positive results than multiplex-nested RT-PCR (Table 3). With results from single RT-PCR as standard, multiplex-nested RT-PCR provided high sensitivity and specificity for amplification of *Pfs25* and *Pvs25.* Furthermore, high values of likelihood ratios of positive test result (82.4 for *Pvs25* to infinity for *Pfs25*) was noted whereas multiplex-nested RT-PCR offered very low values of likelihood ratios of negative test results (Table 4). Both single and multiplex-nested RT-PCR contributed greater efficacy for diagnosing gametocytes of *P. falciparum* and *P. vivax* than microscopy and the performance was more pronounced for those of *P. falciparum* (Table 4).

**Table 4:** **Diagnostic performance of multiplex nested RT-PCR for*Pfs25*and*Pvs25*with single nested RT-PCR as reference**

	**Gametocytes (%)**	
	**P. falciparum**	**P. vivax**
Sensitivity	75/77 (97.4)	81/82 (98.9)
Specificity	80/80 (100)	80/81 (98.8)
Likelihood ratio of positive test	*	82.4
Likelihood ratio of negative test	0.03	0.01
Ratio of single nested RT-PCR positives to	9.63	2.73
microscopy positives		
Ratio of multiplex nested RT-PCR positives to	9.50	2.73
microscopy positives		

* Denominator is zero.

### Transmission seasons and gametocyte prevalence

The prevalence of *P. falciparum* isolates containing gametocytes collected in dry season was not significantly different from that collected in wet season. Likewise, no significant seasonal difference was observed between the prevalence of isolates harbouring *P. vivax* gametocytes. These findings remain the same regardless of the methods (microscopy, single-nested RT-PCR or multiplex-nested RT-PCR) deployed for gametocyte detection (Table 5). However, parasite density of *P. vivax* has significant influence on gametocyte detection by microscopy whereas this effect was not evident for *P. falciparum* (Table 6).

**Table 5:** **Prevalence of*P. falciparum*and*P. vivax*gametocytes among isolates collected in dry and wet seasons**

**Diagnostic method**	**Dry season**		**Wet season**		***p* value**
	**n**	% positive*	**n**	% positive*	
Microscopy					
*P. falciparum* gametocyte	3	6.4	3	7.7	0.81
*P. vivax* gametocyte	17	34	13	32.5	0.88
Single nested RT-PCR					
*P. falciparum* gametocyte	40	85.1	35	89.7	0.52
*P. vivax* gametocyte	46	92	36	90	0.74
Multiplex nested RT-PCR					
*P. falciparum* gametocyte	42	89.4	35	89.7	0.95
*P. vivax* gametocyte	46	92	36	90	0.74
*18S rRNA*PCR					
*P. falciparum*	47	−	39	−	−
*P. vivax*	50	−	40	−	−

* total number of positives for a given species by *18S rRNA* PCR.

- denotes not applicable.

**Table 6:** **Parasite densities of isolates with monoinfections of*P. falciparum*and of*P. vivax*collected in dry and in wet seasons**

**Category**	**Parasite density (parasites/μL)**	
	**Dry season**	**Wet season**
P. falciparum*		
with gametocyte	n = 3	n = 5
range	1560–7000	930–36630
median	2960	6695
no gametocyte	n = 40	n = 27
range	0–288360	0–110840
median	22280	17800
P. vivax*		
with gametocyte	n = 17	n = 11
range	11640–70080 #	6090–49320 #
median	19360	9630
no gametocyte	n = 29	n = 21
range	0–36360	304–16360
median	6560	2740

* Verification of monoinfection by *18S rRNA* PCR.

# Parasite densities between isolates with and without detectable gametocytes by microscopy were significantly different (*p* < 0.001, Mann–Whitney *U* test).

## Discussion

Recent molecular epidemiological surveys of malaria in Thailand have revealed that all five *Plasmodium* species circulate in this country. Most malaria cases are caused by *P. falciparum* (~56.8% of all species) and *P. vivax* (~41.3%). It is noteworthy that co-infections of both these species contributed ~12.5% of all malaria cases; the highest prevalence of ~23–24% occurred along the Thailand and Myanmar borders [18,19]. Therefore, malaria control by transmission-reducing interventions, such as gametocytocidal drugs or sexual stage vaccines, requires simultaneous assessment of human infectious reservoirs of both *Plasmodium* species. The multiplex-nested RT-PCR developed herein offered high diagnostic performance with sensitivity and specificity over 97% when results from single-nested RT-PCR targeting each of these transcripts were used as standard. Multiplex-nested RT-PCR detected gametocytes of *P. falciparum* and *P. vivax* 9.50 and 2.73 times more patient samples, respectively, than was determined by microscopy. It is noteworthy that 87.4% of *P. falciparum* and 91.1% of *P. vivax* clinical isolates in Umpang District contained gametocytes based on detection of *Pfs25* and *Pvs25* mRNA by multiplex-nested RT-PCR and total malaria species identified by *18S rRNA* PCR. Similarly, molecular surveys in Kenya and Tanzania revealed 89.3–92.3% *P. falciparum* gametocyte prevalence despite differences in disease endemicity between these countries and Thailand [22]. The high prevalence of submicroscopic gametocyte carriage has been hypothesized to ensure gametocyte survival by avoiding substantial stimulation for anti-gametocyte immune responses [23,24] and developmental success in vector by preventing damage of mosquito midgut during ookinete invasion through oocyst development [24-26].

A recent study using *P. falciparum* isolates from patients in Tak Province who had febrile symptom ~3 days (range one to eight days) before blood sample collection (n = 44) revealed that all isolates gave positive results for RT-PCR targeting *Pfg377*, a female gametocyte-specific mRNA, whereas microscopy detected gametocytes in only six isolates. Subsequent *in vitro* cultivation resulted in gametocyte production in 89% of isolates as detected by microscopy [27]. Meanwhile, all except one malaria patients in this study had fever one day prior to blood sample collection. Although it is likely that there could be some expression of *Pvs25* and *Pfs25* at the early stages of gametocyte maturation, probably from leaky transcription or low-level transcription, *Pvs25* and *Pfs25* mRNA in blood samples are mainly from mature gametocytes. Taken together, it seems that almost all patients infected with *P. falciparum* in Tak province had mature gametocytaemia within a few days after onset of febrile symptoms *albeit* the majority existing below microscopic detection threshold. Likewise, similar findings were demonstrated in *P. vivax*-infected patients, suggesting that malaria transmission could occur at the beginning or soon after the febrile onset in this endemic area. By contrast, a survey of *P. falciparum* gametocytes among patients in an endemic area in Tak Province by RT-PCR using the method described by Babiker *et al* has shown only 15 of 82 samples (18.3%) contained *Pfs25* mRNA whereas reverse transcriptase-loop-mediated isothermal amplification (RT-LAMP) provided slightly superior results (29.2%) [28]. Discrepancy in prevalence of gametocyte carriage may not be directly compared between studies because of non-identical clinical samples and methodology. However, there remains a possibility that the detection methods conferred different diagnostic sensitivity. Importantly, the minimum diagnostic threshold of the multiplex-nested RT-PCR in this study was 10 copies of templates. Although estimation of detection limit of the multiplex-nested RT-PCR in this study did not directly reflect the actual number of gametocytes in the samples, it is likely that each mature gametocyte of *P. falciparum* or *P. vivax* possesses several copies of *Pfs25* mRNA or *Pvs25* mRNA; thereby a single gametocyte is potentially sufficient to give a positive result.

It has been noted that *P. falciparum* gametocyte carriage is more common in areas with high malaria transmission intensity and the younger age group seems to have higher gametocyte prevalence than in an older age group. However, age-dependent patterns of gametocyte carriage for *P. falciparum* and *P. vivax* have not been documented in this study, consistent with previous findings that in areas with low transmission intensity, age groups of infected individuals seem not to be apparently associated with gametocyte prevalence [29-31]. Meanwhile, the prevalence of gametocyte carriage relative to all malaria cases as detected by either microscopy or multiplex-nested RT-PCR in this study seems to show no significant seasonal fluctuation as previously noted [32,33]. Discrepancy could arise from the small sample size in this study, differences in intensity of malaria exposure among subjects, competency of routine microscopists in diagnosing gametocytes of *P. vivax* or other unknown factors. Nevertheless, the high prevalence of gametocyte carriage detected by multiplex-nested RT-PCR for both *P. falciparum* and *P. vivax* in both high (wet season) and low (dry season) transmission periods could ensure sustainability of malaria endemicity in the study population.

The prevalence of individuals with gametocytaemia based on microscopy showed remarkable spatial variation. However, sensitivity of gametocyte detection by microscopy *per se* could be influenced by the experience of microscopists, the quality of slide preparation and the number of microscopic fields examined [1-3]. Herein, patients who carried microscopically patent *P. vivax* gametocytaemia had significantly higher median parasite density than those without detectable gametocytes by microscopy. Therefore, a higher parasite density may offer a better chance for detecting gametocytes of *P. vivax* prior to anti-malarial treatment. Importantly, a number of factors could influence microscopically patent gametocytaemia, e.g. exposure to anti-malarial drugs, increased in body temperature or anaemia [1]. These constraints, compromising effective assessment of gametocyte carriage, could be alleviated by deployment of RT-PCR-based methods for detecting gametocyte-specific mRNA with remarkably higher sensitivity than conventional microscopy. Because the patterns and dynamics of gametocyte carriage in malaria-infected individuals have important contributions to the capability of malaria transmission and persistence in each endemic area, simultaneous detection of both *P. falciparum* and *P. vivax* gametocytaemia by multiplex-nested RT-PCR developed herein will be useful in areas where both malaria species co-circulate.

## Competing interests

The authors declare that they have no competing interests.

## Authors’ contributions

SJ and CP involved in study design, supervised and managed the project. NK, CP, UP and SJ carried out field work. NK, SJ and CP carried out molecular assay. NK and UP performed microscopy-based detection and estimation of parasite density. NK, CP and SJ analysed data. SJ and CP wrote the manuscript. All authors read and approved the final manuscript.
